# Person and Family Centredness—The Need for Clarity of Focus

**DOI:** 10.3390/ebj5020014

**Published:** 2024-05-27

**Authors:** Brendan McCormack

**Affiliations:** 1 The Susan Wakil School of Nursing and Midwifery (Inc. Sydney Nursing School), Faculty of Medicine and Health, The University of Sydney, Sydney 2006, Australia; brendan.mccormack@sydney.edu.au; 2 Faculty of Health Sciences, The University of Maribor, Žitna ulica 15, 2000 Maribor, Slovenia; 3 School of Nursing and Paramedic Sciences, Ulster University, 2-24 York Street, Belfast BT15 1AP, UK; 4 Department of Oncology and Palliative Care, Zealand University Hospitals, Vestermarksvej 9, 4000 Roskilde, Denmark; 5 Faculty of Health Sciences, University of Southern Denmark, 5230 Odense, Denmark

Congratulations to the editorial team of the *European Burn Journal* for having the vision to host a Special Issue on the theme of “Person-Centred and Family-Centred Care Following Burn Injuries”. Whilst in many ways it would be fabulous if we were at the stage of global healthcare where person-centredness was the ‘norm’ in terms of practice and if editorials and Special Issues such as this one were no longer necessary, it is fair to say that we are a long way from that reality, and we continue to exist in dominant technico-rational models of care that are heavily influenced by the philosophy of new public management with the primacy of the 3Es—economy, efficiency and effectiveness. Of course, in a period of challenging global economic conditions, the need for a focus on the 3Es is important, but what is equally important is to never lose sight of the fact that healthcare is about persons, in all our complexity!

I do not proport to be a burns expert, but like many conditions that have a profound effect on the human condition, a person sustaining a burn often experiences a life changing situation that requires careful and holistic treatment and care. Also like many traumatic injuries, burns traverse the whole trajectory of care from emergency through to long-term rehabilitation and care, which for some persons continues for the remainder of their lives. So, given the significant trauma associated with burns, it seems imperative that a person-centred and whole-system approach is adopted, thus demonstrating why this Special Issue is important and significant.

Of course, whilst welcoming this Special Issue, it is also the case that the papers themselves represent an eclectic mix of foci on burn care and treatment. Like many published papers that espouse a person-centred focus, few if any authors define what they mean by person-centred care or practice or provide a conceptual or theoretical framework for framing their research. Further, like in the literature in general, we see the interchangeable use of terms such as patient and person-centred. Whilst some authors suggest that we should not get too ‘hung up’ on clarity of definitions and terms (Mitchell et al., 2022) [1] as it may limit the potential of the scholarship in the field of person-centred healthcare, it is my contention that one of the reasons why person-centred healthcare has not evolved to the extent it should have is precisely because of a lack of precision in what is referred to as person-centred healthcare/practice. Further, whilst sustaining a burn is seen as a family injury, requiring a family-centred approach to the provision of care and treatment, the authors of only one paper in this Special Issue state clearly what they mean by ‘family-centred care’ (Bayuo & Agbeko 2024) [2]. This in itself raises questions about the use of the term ‘family-centred’ in this context—family is a collection of connected persons, so how do we respect the personhood of family members as persons in their own right? Does the focus on the family as a unit override the personhood of individual family members? What is the balance between treating and caring for individual family members vis-à-vis the treatment of the family as a single unit? These are significant questions and raising them, highlights the importance of being clear about the terms we use in care provision, as these terms have implications for how care is provided and indeed if it can claim to be person- and/or family-centred.

I have highlighted ‘personhood’ as an important consideration when we lay claim to person-centredness. Whilst the philosophy of personhood is complex and multifaceted, I contend that ‘authenticity’ is a unifying principle that holds a person’s beliefs, values, hopes, dreams and desires as central considerations in how we provide help to another person. Think about our closest friendships and relationships—in these relationships we share our deepest sense of ‘self’ with others, enabling others to know who we are as people, what values are important to us, the dreams, hopes and desires we hold in our lives and the kind of life that we strive to live. These are all key considerations in treating and caring for persons impacted by a burn injury and in knowing them as a person, so that treatment and care can be truly individualised. Knowing the person in this way is essential to enacting personhood, and knowing how to maximise a person’s autonomy is a key consideration in person-centred practice, i.e., how do we as healthcare workers ensure that the person in focus has as much control over decision-making as possible? Having some sense of what authenticity means to a person and how that manifests itself through their being-in-the-world is essential to working in a person-centred way. Through our discussions, reflections, debates, arguments and agreements, our knowing of the person is shaped and reshaped, ordered and reordered, prioritized and reprioritized as life progresses. It is in this nexus of relationships between persons that we need to consider how the dynamic of person-centred and family-centred care plays out. How do you respect the autonomy of the patient as person with the collective needs of the family?

Throughout life, persons continually grow, develop and experience transition and so there is always the potential for a new direction in life to be taken and so we should never assume that family can act as a proxy for individual patient decision-making. Striving to know the patient as an authentic person is critical to how we balance individual with family decision-making. Reflections on the effectiveness of decisions are always considered in the context of knowing a person and the kind of authentic choices they would make. For healthcare workers to facilitate authentic decision-making, we need to know what sorts of things are important to the person, we must evaluate the relative intensity and reality of their desires, we must order their preferences consistently and we must envisage and provide alternative plans for action. But of course, facilitating deliberation on care decisions is not undertaken in isolation from others, but instead requires engagement, collaboration, reflection and imagination. Meyers (1989) [3] suggests that persons’ require both ‘resistance’ and ‘resolve’ to carry out their desired plans—resistance of unwarranted pressure from other persons and resolve in determination to act on their own judgements. Nurses should never take for granted the knowledge, skills and expertise needed to do this well and the privileged position we have in helping a person (who has sustained a burn injury) make meaningful plans for their future.

Such a privilege cannot be taken for granted by healthcare teams and healthcare systems. Too often, the rhetoric of person-centred care is espoused in healthcare strategy and policy. Executive leaders write it into their vision statements and organisational philosophies bestow the virtues of person-centredness in shaping how the organisation functions. But for many clinicians, the gap between this espoused philosophy and the lived experience of providing care and treatment on a daily basis can be very different. For over 30 years, I have postulated, supported by evidence, that organisations/healthcare systems cannot demand person-centred care for patients and families without a clear responsibility for developing and sustaining person-centred cultures. In my view, it is immoral for an organisation to expect high-quality, evidence-informed, person-centred care to be provided to patients and families without an equal focus on the personhood of staff and their wellbeing (McCormack & McCance 2017) [4]. I have challenged the dominant focus on person-centred care provided to, with and for patients/families at the expense of staff wellbeing. This values-informed position has been reinforced through the systematic development, implementation and evaluation of the Person-centred Nursing Framework (PCNF) (McCormack & McCance 2021) [5] and the Person-centred Practice Framework (PCPF) (McCance & McCormack 2021) [6], that have shaped much of my work in person-centred healthcare. It remains the case though that the person-centredness of care providers is placed on a lower level of importance and significance to that of patients/families—and this has been abundantly evident during the COVID-19 pandemic.

For person-centredness to become more normalised in healthcare systems, there is a need for thought-leaders, strategic planners, managers and decision-makers to stop defending organisational cultures that are non-conducive to the achievement of person-centred care for patients and families but instead strive to make sense of what person-centredness could look like for all persons in an organisation. For too many managers, an obsession with ‘quick-fix’ solutions mean that the time needed to achieve such clarity is sacrificed at the altar of expediency. Person-centred practice needs to be understood as a concept that is embedded in every strategy and policy that shapes healthcare planning and delivery. It needs to be based on conceptual and theoretical frameworks that are inclusive of all persons and that clearly articulate how these concepts are to be embedded in everyday practices at macro-, mezzo- and micro-levels.
